# Epigenetic regulation of *AXL* and risk of childhood asthma symptoms

**DOI:** 10.1186/s13148-017-0421-8

**Published:** 2017-11-07

**Authors:** Lu Gao, Joshua Millstein, Kimberly D. Siegmund, Louis Dubeau, Rachel Maguire, Frank D. Gilliland, Susan K. Murphy, Cathrine Hoyo, Carrie V. Breton

**Affiliations:** 1 Department of Preventive Medicine, USC Keck School of Medicine, 2001 N. Soto Street, Los Angeles, CA 90032 USA; 2 Department of Biological Sciences, Center for Human Health and the Environment, North Carolina State University, Raleigh, NC 27695 USA; 3 Division of Gynecologic Oncology, Department of Obstetrics and Gynecology, Duke University School of Medicine, Durham, NC 27710 USA

**Keywords:** Methylation, Children, Epigenetics

## Abstract

**Background:**

*AXL* is one of the TAM (*TYRO3*, *AXL* and *MERTK*) receptor tyrosine kinases and may affect numerous immune-related health conditions. However, the role for *AXL* in asthma, including its epigenetic regulation, has not been extensively studied.

**Methods:**

We investigated the association between *AXL* DNA methylation at birth and risk of childhood asthma symptoms at age 6 years. DNA methylation of multiple CpG loci across the regulatory regions of *AXL* was measured in newborn bloodspots using the Illumina HumanMethylation450 array on a subset of 246 children from the Children’s Health Study (CHS). Logistic regression models were fitted to assess the association between asthma symptoms and DNA methylation. Findings were evaluated for replication in a separate population of 1038 CHS subjects using Pyrosequencing on newborn bloodspot samples. *AXL* genotypes were extracted from genome-wide data.

**Results:**

Higher average methylation of CpGs in the *AXL* gene at birth was associated with higher risk of parent-reported wheezing, and the association was stronger in girls than in boys. This relationship reflected the methylation status of the gene-body region near the 5′ end, for which a 1% higher methylation level was significantly associated with a 72% increased risk of ever having wheezed by 6 years. The association of one CpG locus, cg00360107 was replicated using Pyrosequencing. Increased *AXL* methylation was also associated with lower mRNA expression level of this gene in lung tissue from the Cancer Genome Atlas (TCGA) dataset. Furthermore, *AXL* DNA methylation was strongly linked to underlying genetic polymorphisms.

**Conclusions:**

*AXL* DNA methylation at birth was associated with higher risk for asthma-related symptoms in early childhood.

**Electronic supplementary material:**

The online version of this article (10.1186/s13148-017-0421-8) contains supplementary material, which is available to authorized users.

## Background

Asthma is the most common chronic disease in childhood [1, 2]. It is a complex disease determined by the interplay between genetic and environmental factors [3–7]. The pathogenesis of childhood asthma is characterized by both structural features in the airway wall, such as breach in epithelium integrity, and immunological features like airway inflammation [8–11]. We and others have shown that early life exposure to tobacco smoke and air pollution are associated with increased risk of childhood asthma and asthma symptoms [12–17]. In addition, epigenetic modifications, including DNA methylation, can alter regulation of genes involved in airways development or immune-mediated inflammatory pathways and may play a role in mediating the effects of environmental exposures [18–22]. Many studies have now been conducted to investigate the effects of epigenetic variation on risk of asthma-related phenotypes [23, 24]. Although alterations in DNA methylation patterns can occur throughout life, important patterns in the methylome are established during embryogenesis and early life [25]. However, few studies have examined the effects of DNA methylation in immune cells at birth on asthma pathogenesis.

In previous work, we identified a gene—*AXL*, one of the TAM (*TYRO3*, *AXL* and *MERTK*) family receptor tyrosine kinases—in which methylation status of certain CpG loci varied based on prenatal exposure to tobacco smoke [26, 27]. Given the known associations between prenatal tobacco smoke exposure and asthma risk, as well as prenatal tobacco smoke and DNA methylation in *AXL*, we sought to investigate whether methylation in *AXL* at birth was associated with childhood asthma or asthma-related symptoms.

TAM genes are key signaling molecules in innate immune responses and may affect numerous immune-related health conditions [28]. They act as pivotal inhibitors of immuno-regulatory factors and prevent unrestrained signaling of inflammatory responses by these factors [29, 30]. Growth-arrest-specific 6 (*GAS6*) and protein S are the ligands that bind and activate the TAM receptors [31], and *GAS6* showed higher expression in subjects with severe asthma during exacerbation [32]. Moreover, genetic polymorphisms in TAM genes and their ligands have also been implicated in inflammation and autoimmune diseases [33, 34]. Genetic variants may affect DNA methylation of CpG sites in their genomic surroundings and gene expression through altering the affinity of DNA binding factors, enhancer activity, or chromatin formation [35, 36]. Increased CpG methylation in the promoter region may modulate expression or silence *AXL* entirely and lead to overstimulation of the immune system. The identification of epigenetic and genetic variation associated with childhood asthma symptoms may shed light on the etiology of this complex disease and the biological role of *AXL*.

In this study, we investigated the association between methylation of multiple CpG sites across the regulatory regions of *AXL* at birth and risk of childhood asthma symptoms, taking into consideration the underlying genetic variation in *AXL*. We first assessed the association in a subset of 246 subjects from the Children’s Health Study (CHS), then sought to replicate the associations in a separate population of 1038 CHS subjects. Correlations between DNA methylation and expression of *AXL* were also evaluated in two tissue types, cord blood from 235 subjects of the Newborn Epigenetic STudy (NEST) and lung tissue samples from the Cancer Genome Atlas (TCGA) dataset [37–39]. To further investigate the epigenetic control of *AXL* and address potential confounding effects from genetic polymorphisms, the association between *AXL* genetic polymorphisms and methylation was also evaluated.

## Methods

### Study population

This study was conducted in subsets of participants in the Children’s Health Study, a longitudinal study of respiratory health of children in southern California [6, 40–42]. A subset of 737 children were initially sampled to participate in a study of atherosclerosis [43] of whom 689 could be linked to California birth records. Of these, we randomly selected 246 children from participants for whom at least 700 ng of DNA was available from a dried newborn bloodspot. DNA methylation was assessed in the newborn bloodspots using the Infinium HumanMethylation450 BeadChip (HM450) arrays. A separate subset of 1038 CHS subjects who were not participants in the atherosclerosis study, but who were enriched in children with asthma, was selected to have newborn bloodspot DNA methylation measured by Pyrosequencing. By design, neither population had exposure to *in utero* tobacco smoke.

Personal, parental, socio-demographic characteristics including maternal smoking during pregnancy and medical history for all CHS subjects were obtained from parent-completed questionnaires at study entry. Asthma and related symptoms at age 6 years were evaluated through these questionnaires and included (a) asthma (defined by a “yes” answer to the question “Has a doctor ever diagnosed this child as having asthma?”); (b) wheeze (defined by a “yes” answer to the question “Has your child’s chest ever sounded wheezy or whistling?”); and (c) wheeze in the previous 12 months; (d) bronchitic symptoms in the previous 12 months (defined by the parent’s report of a daily cough for 3 months in a row, congestion of phlegm other than when accompanied by a cold, or bronchitis).

A subset of 235 Newborn Epigenetics STudy (NEST) subjects was evaluated for the association between methylation at several *AXL* CpG loci and its mRNA level in cord blood. The NEST is a prospective study of women and their children [44]. It was designed to identify exposures during pregnancy and early life associated with stable epigenetic alterations in infants that may alter chronic disease susceptibility later in life. Women were eligible if they were aged 18 years and older, were pregnant, and spoke English. The catchment area for Duke Maternal Fetal Medicine prenatal care clinic largely includes three contiguous counties in central North Carolina (NC): Durham, Orange, and Wake. Women who met eligibility criteria were either consented and interviewed in-person in consultation rooms during the visit or given the questionnaire to self-administer and mail back to the study office. Smokers were preferentially enrolled to the extent possible, identified through medical records.

### DNA methylation

**Fig. 1:**
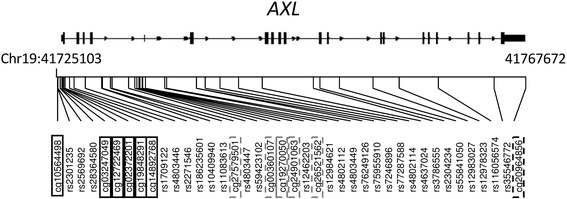
Genomic location of *AXL* CpG sites and SNPs under investigation. Solid black box: CpG sites in the near-TSS region (cg10564498, cg03247049, cg12722469, cg02372201, cg19848291 and cg14892768); dashed gray box: CpG sites in the gene-body region (cg27579501, cg00360107, cg19270050, cg24901063, and cg26521562); dashed black box: CpG site in the 3′ untranslated region (cg20964856). TSS = transcription start site

DNA methylation was measured in newborn bloodspots (NBS) that were obtained as part of the routine California Newborn Screening Program from the California Department of Public Health Genetic Disease Screening Program. The NBS were stored by the state of California at − 20 °C. A single complete newborn bloodspot for each requested participant was mailed to us and then stored in our lab at − 80 °C upon receipt. Laboratory personnel performing DNA methylation analysis were blinded to study subject information. DNA was extracted from whole blood cells using the QiaAmp DNA blood kit (Qiagen Inc., Valencia, CA) and stored at − 80 °C. Seven hundred nanograms to 1 μg of genomic DNA from each sample was treated with bisulfite using the EZ-96 DNA Methylation Kit™ (Zymo Research, Irvine, CA, USA), according to the manufacturer’s recommended protocol and eluted in 18 μl. The Infinium HM450 data was compiled for each locus and was expressed as beta (*β*) values. Minfi package (version 1.16.0) in R was used to process the HM450 array data [45], applying a normal exponential background correction to the raw intensities to reduce array-level background noise followed by dye-bias correction [46]. We then normalized each sample’s methylation values to the same quantiles to address sample-to-sample variability [47]. Seven cord blood cell sub-populations (CD8+ T-lymphocytes, CD4+ T-lymphocytes, natural killer cells, B-lymphocytes, monocytes, granulocytes, and nucleated red blood cells) were estimated using regression calibration approach algorithm described by Bakulski et al. [48, 49]. After preprocessing, CpG loci containing single-nucleotide polymorphisms (SNPs) were removed from analyses. DNA methylation was studied for a total of 12 features on the HM450 array spanning the *AXL* gene, identified according to their genomic positions (Fig. 1).

For Pyrosequencing assays, three CpG loci (cg10564498, cg12722469, and cg00360107) were selected for replication based on results in the primary population. For NEST subjects, genomic DNA from buffy coat specimens was extracted from umbilical cord blood using Puregene Reagents (Qiagen, Valencia, CA). PCR primers were designed by EpigenDx Inc. (http://www.epigendx.com) to cover the loci of interest, and the specificity of the primer sequences was confirmed using in silico PCR. Five hundred nanograms of genomic DNA extracted from NEST and CHS samples (randomized together) was bisulfite treated using the EZ DNA Methylation Kit™ (Zymo Research, Irvine, CA, USA) and was purified according to the manufacturer’s protocol. Methylation assays (assay ADS8097-FS) were performed by EpigenDx Inc. using the PSQ96HS system (Pyrosequencing, Qiagen) according to standard procedures as described in previous work [50, 51]. The methylation level was determined using QCpG software (Pyrosequencing, Qiagen) and was reported as percent of DNA methylation at each CpG locus. Each experiment included cytosines not part of a CpG dinucleotide as internal controls to evaluate incomplete bisulfite conversion of the input DNA. A series of unmethylated and methylated DNA were included as controls in each assay. Furthermore, PCR bias testing was performed by mixing unmethylated control DNA with in vitro methylated DNA at different ratios (0, 5, 10, 25, 50, 75, and 100%), followed by bisulfite modification, PCR, and Pyrosequencing analysis.

### mRNA expression in NEST

Origene’s qStar mRNA detection system (Origene, Rockville, MD) was used in the quantification of *AXL* mRNA in cord blood in NEST subjects. qPCR primers for the major *AXL* transcript (#HK228780) and its corresponding copy number standard (#HK201002) were designed by qStar. All measurements of expression were conducted in duplicate in cord blood samples from 235 participants in the NEST cohort. *AXL* mRNA was isolated from stored PAXgene tubes of cord blood using the PAXgene blood miRNA isolation kit (Qiagen, Valencia, CA). First strand cDNA conversion of mRNA was performed using Origene’s cDNA synthesis kit (#NP100042). qPCR reactions were run with Kappa Sybr Fast qPCR kit (# KK4604; KapaBiosystems, Boston, MA) in the ABI 7900HT thermocycler (Thermofisher). Ten percent repeats were included to evaluate reproducibility.

### In silico analyses in publicly available data

To assess the association between DNA methylation and gene expression in lung tissue, we downloaded *AXL* methylation profiling data of 29 histologically normal tissue samples from cases with lung adenocarcinoma (LUAD) or lung squamous cell carcinoma (LUSC) from the TCGA dataset [37, 39]. All samples had both methylation profiling (Illumina Infinium HumanMethylation450 Beadchip) and RNA-seq (Illumina HiSeq) data. The mean age was 65.9 years (SD 12.39), and 75.9% of the subjects were male. 51.7% of the subjects were moderate to heavy smokers.

To visualize epigenetics marks and regulatory regions of the *AXL* gene in relevant tissues and cell types, we used the WashU EpiGenome Browser [52, 53].

### Genotyping

Buccal scrapes were collected from CHS subjects beginning in 1998 using standard protocols [54]. A customized package including three buccal kits with instructions on buccal cell collection was sent to each participant. Genomic DNA was isolated from buccal cells using a Puregene™ DNA isolation Kit (Gentra Systems, Minneapolis, MN), and genotyping was performed using the Illumina HumanHap550, HumanHap550-Duo, or Human610-Quad BeadChip microarrays as described previously [55]. Data was phased using SHAPEIT and imputed using IMPUTE2 with 1000 Genomes Phase 1 integrated variant v3 phased reference (April 2012). Genotypes of SNPs in *AXL* and its surrounding region (1 kb upstream and downstream) were extracted from the CHS genome-wide genotypic data. SNPs with minor allele frequency (MAF) less than 5% or missing in more than 5% of the samples were removed, leaving 90 SNPs for analyses. RS numbers, minor allele frequencies, and genomic locations of all 90 SNPs under investigation were shown in Additional file 1: Table S1. Twenty-eight tagged SNPs were identified with a pair tag *r* ^2^ > 0.8 in Haploview using all available CHS samples (*N* = 3845) and were included in the analyses [56]. In addition, we performed principal component (PC) analysis on the 28 *AXL* tagged SNPs, and the top 7 PCs, which represented 80% of the total variation cumulatively, were added as covariates in regression models to test confounding effects from gene polymorphisms. Genotype data was available for 207 of the 246 subjects in the primary population and for 728 of the 1038 subjects in the replication study. Admixture was assessed using the program STRUCTURE from a set of ancestral informative markers that were scaled to represent the proportion of African American, Asian, Native American, and white admixture [57].

### Statistical analyses

Descriptive analyses were performed to examine the distribution of methylation and subject characteristics. Spearman correlations of methylation between each CpG site were calculated and shown in Additional file 1: Table S2. We took the average of methylation at CpG sites in the same genomic region to represent regional methylation status (Fig. 1). To evaluate the association between *AXL* methylation and asthma symptoms, we fitted logistic regression models for each outcome and CpG individually, adjusted for child’s age, sex, ethnicity, city of residence at study entry, and plate effect, while history of doctor-diagnosed asthma was additionally adjusted for wheezing and bronchitic outcomes. Additional adjustment for genetic polymorphisms, methylation slide, estimated cord blood cell type proportions, parental education level, allergy history, birth weight, mode of delivery, gestational age, environmental exposures (pets, pests, cockroaches, mildew and carpet), asthma medication use, and admixture did not change the effect estimates by more than 10% and were removed from final models. The results of sensitivity analyses assessing the confounding effects of admixture and top 7 PCs from *AXL* SNPs were shown in Additional file 1: Table S4 and Table S5, respectively. Confounding effects from each of the 28 *AXL* tagged SNPs were also tested one by one and were found to be minimal (results not shown). To estimate if the associations between *AXL* methylation and asthma symptoms were modified by sex, we included an interaction term between sex and methylation in the regression models. Wald tests were used to compute interaction *p* values.

A similar logistic regression model was used in the replication population to evaluate the association between asthma symptoms and methylation at each of the three CpG sites (cg10564498, cg12722469, and cg00360107) measured by Pyrosequencing, with adjustment for child’s age, sex, ethnicity, city of residence at study recruitment, and asthma history (for wheezing and bronchitic outcomes). Adjusting for methylation plate had no effect on results and was not included in the final model. Effect modification by sex was also assessed.

Linear regression models were used to evaluate the associations between genetic polymorphisms and DNA methylation at *AXL* CpG sites, adjusting for sex, admixture, and gestational age. All SNPs were coded additively by the number of minor alleles. Logistic regression models were used to assess the association between *AXL* SNPs and asthma symptoms, adjusting for child’s age, sex, ethnicity, and admixture. We controlled the false discovery rate (FDR) at the 0.05 level using the Benjamini-Hochberg procedure [58], accounting for multiple tests across CpG sites and SNPs in *AXL*.

All tests assumed a two-sided alternative hypothesis and were conducted using the R programming language, version 3.3.1.

## Results

### DNA methylation of *AXL* and risk of asthma symptoms

Demographic characteristics of the primary and replication study populations are shown in Table 1. The primary population had fewer males, more Hispanic subjects, and lower parental education level. There were more subjects having doctor-diagnosed asthma and related symptoms in the replication population by design. Prevalence of asthma was 16% in the primary study population and 28% in the replication population. Participants were 6 years old on average in the primary population and 7 years old in the replication population at the time of asthma symptoms assessment. Many of the 12 CpG loci were significantly correlated (Additional file 1: Table S2), with CpG sites closer in proximity showing stronger correlations.

**Table 1:** Demographic characteristics of participants

	Primary study population (*N* = 246)	Replication population (*N* = 1038)	*p* value^a^
Male sex, *n* (%)	98 (39.8)	541 (52.1)	0.0005
Ethnicity, *n* (%)			0.05
Hispanic	147 (59.8)	531 (51.3)	
Non-Hispanic White	72 (29.3)	381 (36.8)	
Asian/Black/Other	27 (11.0)	124 (12.0)	
Ever MD-diagnosed asthma, *n* (%)	39 (15.9)	295 (28.4)	< 0.0001
Ever wheezing, *n* (%)	66 (26.8)	455 (43.8)	< 0.0001
Wheezing in the previous 12 months, *n* (%)	37 (15.0)	250 (24.1)	0.001
Bronchitic symptoms in the previous 12 months, *n* (%)	40 (16.3)	229 (22.1)	0.04
Parental education, *n* (%)			0.002
High school or less	89 (36.6)	316 (31.3)	
Some college	76 (31.3)	438 (43.5)	
Finished college/some graduate school	78 (32.1)	254 (25.2)	
Age years, mean (sd)	6.4 (0.6)	7.2 (1.3)	< 0.0001
Gestational age days, mean (sd)	277.5 (11.0)	272.7 (11.3)	< 0.0001

^a^Derived from a Pearson’s Chi-squared test for categorical variables and from an unequal variance 2-sample *t* test for continuous variables

We first investigated whether average DNA methylation in *AXL* was associated with childhood asthma symptoms (Table 2). Average methylation of all 12 CpG sites was positively associated with ever wheezing (OR 1.46, 95% CI 1.12–1.91), and the association remained significant after adjusting for multiple testing at these genomic regions (FDR-adjusted *p* value 0.008). This was mainly driven by methylation status of the gene-body region near the 5′ end, for which a 1% higher methylation level was significantly associated with a 72% higher risk of ever wheezing (OR 1.72, 95% CI 1.30–2.28) and a 109% higher risk of wheezing in the previous 12 months (OR 2.09, 95% CI 1.32–3.30). Moreover, the effects of average *AXL* methylation on risk of wheezing in the previous 12 months were limited to girls (OR 1.88, 95% CI 1.09–3.24) and not boys (OR = 0.75, 95% CI 0.40–1.39; *p*_int_ = 0.03). Increased *AXL* methylation was also associated with higher risk for acute bronchitic symptoms, although effects were not significant.

**Table 2:** Association between average DNA methylation levels at *AXL* CpG sites and risk of asthma and related symptoms in childhood in the primary study population (*N* = 246)

	Average of near-TSS CpG sites^a^		Average of gene-body CpG sites^b^		Average of all 12 CpG sites^c^	
	OR	*p*	OR	*p*	OR	*p*
Ever MD-diagnosed asthma						
Overall	1.10	0.16	1.05	0.71	1.17	0.19
By sex						
Boys	1.17	0.10	1.12	0.58	1.34	0.09
Girls	1.03	0.79	1.00	1.00	1.02	0.90
Interaction *p* value	0.33		0.69		0.28	
Ever wheezing						
Overall	1.10	0.19	1.72	*0.0002*	1.46	*0.005*
By sex						
Boys	1.06	0.60	2.70	*0.001*	1.42	0.12
Girls	1.12	0.20	1.51	*0.01*	1.48	*0.02*
Interaction *p* value	0.69		0.08		0.88	
Wheezing in the previous 12 months						
Overall	0.97	0.79	2.09	*0.002*	1.26	0.25
By sex						
Boys	0.73	0.10	1.85	0.11	0.75	0.35
Girls	1.16	0.31	2.20	*0.005*	1.88	*0.02*
Interaction *p* value	*0.05*		0.70		*0.03*	
Bronchitic symptoms in the previous 12 months						
Overall	0.99	0.90	1.23	0.14	1.04	0.74
By sex						
Boys	0.94	0.51	1.18	0.46	0.92	0.64
Girls	1.05	0.63	1.26	0.19	1.18	0.35
Interaction *p* value	0.41		0.82		0.32	

The primary study population (*N* = 246) is adjusted for child’s age, sex, ethnicity, methylation plate and city of residence at study recruitment; additionally adjusted for ever had MD-diagnosed asthma for wheezing and bronchitic outcomes. Odds ratios are presented for an increase in 1% of DNA methylation level at birth. For all comparisons, the reference group is children not having the corresponding outcome. Significant *p* values (< 0.05) are marked in italics

*TSS* transcription start site

^a^Average of cg10564498, cg03247049, cg12722469, cg02372201, cg19848291 and cg14892768

^b^Average of cg27579501, cg00360107, cg19270050, cg24901063 and cg26521562

^c^Average of all 12 CpG sites

We sought to replicate results of four individual CpG loci in the primary analysis (Table 3 and Additional file 1: Table S3). We chose the two loci in the gene body with the most consistent associations (cg00360107 and cg19270050) and the two loci showing significant interactions with sex (cg10564498 and cg12722469) (Additional file 1: Table S3). These loci were evaluated in a separate population of 1038 CHS subjects using Pyrosequencing. A successful PCR primer design could not be found for cg19270050; therefore, only cg00360107, cg10564498, and cg12722469 were evaluated in the replication population (Table 3). Consistent with primary results, methylation at cg00360107 was also negatively associated with asthma-related symptoms, especially the risk of ever wheezing (Table 3; OR 0.90, 95% CI 0.82–0.99). The differences in associations by sex were marginally replicated for cg10564498 (*p*_int_ = 0.06), but not for cg12722469 (Table 3). In both populations, higher cg10564498 methylation was associated with higher risk for ever wheezing and wheezing in the previous 12 months in girls but lower risk in boys, with similar magnitudes of sex-stratified effects.

**Table 3:** Association between DNA methylation levels at selected *AXL* CpG sites and risk of asthma and related symptoms in childhood in the primary study population (*N* = 246) and replication population (*N* = 1038)

	cg10564498				cg12722469				cg00360107			
	Primary study population		Replication population		Primary study population		Replication population		Primary study population		Replication population	
Distance to TSS (bp)	− 455		− 455		− 55		− 55		6826		6826	
Mean methylation (%)	26.71		19.60		17.05		11.32		6.68		4.35	
	OR	*p*	OR	*p*	OR	*p*	OR	*p*	OR	*p*	OR	*p*
Ever MD-diagnosed asthma												
Overall	1.02	0.28	1.00	0.86	1.05	0.24	0.99	0.72	0.73	*0.04*	0.99	0.86
By sex												
Boys	1.04	0.11	1.01	0.60	1.08	0.24	0.99	0.73	0.72	0.16	1.02	0.76
Girls	0.99	0.80	0.98	0.39	1.03	0.59	0.99	0.87	0.73	0.13	0.96	0.54
Interaction *p* value	0.24		0.32		0.59		0.92		0.94		0.51	
Ever wheezing												
Overall	1.03	0.21	0.97	0.13	1.07	0.09	0.95	*0.05*	0.78	0.07	0.90	*0.04*
By sex												
Boys	0.98	0.59	0.92	*0.006*	1.07	0.38	0.92	*0.01*	1.05	0.87	0.92	0.20
Girls	1.07	*0.04*	1.02	0.44	1.08	0.14	0.99	0.86	0.69	0.04	0.89	0.10
Interaction *p* value	0.09		*0.01*		0.94		0.10		0.20		0.73	
Wheezing in the previous 12 months												
Overall	1.02	0.60	0.96	*0.03*	0.99	0.93	0.93	*0.007*	0.55	*0.04*	0.95	0.34
By sex												
Boys	0.92	0.13	0.92	*0.004*	0.82	0.10	0.92	*0.02*	0.74	0.37	0.90	0.12
Girls	1.15	*0.02*	1.00	0.94	1.11	0.22	0.95	0.16	0.40	*0.03*	1.03	0.68
Interaction *p* value	*0.009*		0.06		*0.04*		0.58		0.23		0.19	
Bronchitic symptoms in the previous 12 months												
Overall	0.99	0.66	0.95	*0.02*	1.06	0.19	0.95	*0.05*	0.75	0.07	0.93	0.15
By sex												
Boys	0.96	0.24	0.95	*0.04*	1.02	0.74	0.94	0.07	0.80	0.31	0.93	0.24
Girls	1.03	0.46	0.97	0.25	1.09	0.14	0.97	0.37	0.70	0.12	0.93	0.40
Interaction *p* value	0.18		0.60		0.47		0.60		0.67		0.96	

The primary study population (*N* = 246) is adjusted for child’s age, sex, ethnicity, methylation plate and city of residence at study recruitment; additionally adjusted for ever had MD-diagnosed asthma for wheezing and bronchitic outcomes

The replication population (*N* = 1038) is adjusted for child’s age, sex, ethnicity and city of residence at study recruitment; additionally adjusted for ever had MD-diagnosed asthma for wheezing and bronchitic outcomes

Odds ratios are presented for an increase in 1% of DNA methylation level at birth. For all comparisons, the reference group is children not having the corresponding outcome. Significant *p* values (< 0.05) are marked in italics

*TSS* transcription start site

### *AXL* methylation and expression in cord blood and lung

Next, we sought to identify whether DNA methylation in *AXL* was associated with its mRNA expression level in the cord blood and in the lung tissue. To do so, we evaluated the correlations between paired data in NEST and TCGA datasets. While transcripts of *AXL* mRNA were detectable in the cord blood in NEST, overall expression was very low and we did not find evidence to support a correlation. We then evaluated the correlations with gene expression for *AXL* methylation using 29 histologically normal lung tissue samples based on HM450 array and RNA sequencing data (Additional file 1: Figure S1). Average methylation of the whole *AXL* gene as represented by 12 CpG loci, showed negative correlation with expression (*r* = − 0.42, *p* value = 0.03). These data, albeit in a population of adult males some of whom have a history of smoking, lend preliminary support to the notion that increased methylation may lead to lower *AXL* expression level in the lung, a more pathologically relevant tissue for asthma and related phenotypes than evaluation of peripheral blood.

### Genetic variants and DNA methylation of *AXL*

We also tested whether SNPs in *AXL* and the surrounding regions (1 kb upstream and downstream) were associated with average DNA methylation in the primary study population (Table 4). A few tagging SNPs were significantly associated with average *AXL* DNA methylation in the near-transcription start site (TSS) region and the whole gene after FDR adjustment. We further tested if SNPs were associated with DNA methylation at individual CpG sites in the replication population and found that *AXL* DNA methylation was strongly linked to underlying genetic polymorphisms (Table 5). The associations between cg10564498 methylation and tagging SNPs are shown in Additional file 1: Figure S2, suggesting that SNPs were having stronger associations with CpG sites in closer proximity. The SNPs under investigation were tagging SNPs; thus, linkage disequilibrium (LD) was low by design (Additional file 1: Figure S2). None of these SNPs were confounders to the association between *AXL* methylation and asthma-related symptoms (Additional file 1: Table S5) or statistically significantly associated with asthma and related symptoms in childhood (Additional file 1: Table S6).

**Table 4:** Association between DNA methylation levels at multiple CpG sites and gene polymorphisms in *AXL* in the primary study population (*N* = 207)

		Average of near-TSS CpG sites^a^			Average of gene-body CpG sites^b^			Average of all 12 CpG sites^c^		
RS Number	Location	*β*	*p*	Adjusted *p*	*β*	*p*	Adjusted *p*	*β*	*p*	Adjusted *p*
rs2301235	41724671	0.60	0.14	0.29	0.09	0.64	0.79	0.35	0.11	0.26
rs2569692	41724687	1.55	2.9E−04	*0.01*	0.24	0.24	0.44	0.84	2.1E−04	*0.01*
rs28364580	41724885	1.16	0.005	*0.05*	0.34	0.07	0.22	0.67	0.002	*0.03*
rs1709122	41725754	1.21	4.2E−04	*0.01*	0.05	0.76	0.87	0.59	0.001	*0.03*
rs4803446	41726167	0.66	0.09	0.25	0.22	0.23	0.44	0.41	0.05	0.19
rs2271546	41727197	0.87	0.12	0.28	0.46	0.08	0.24	0.63	0.03	0.16
rs186235601	41728703	1.34	0.05	0.22	0.52	0.11	0.26	0.89	0.02	0.10
rs10409940	41728765	0.78	0.03	0.16	− 0.06	0.73	0.86	0.33	0.09	0.25
rs11083613	41729505	0.03	0.93	0.95	− 0.33	0.06	0.22	− 0.18	0.38	0.56
rs4803447	41731175	1.75	0.009	0.08	0.52	0.10	0.25	1.01	0.004	**0.05**
rs59423102	41731749	− 0.13	0.81	0.89	0.22	0.38	0.56	0.07	0.81	0.89
rs12462203	41732423	0.18	0.59	0.75	− 0.05	0.74	0.87	− 0.02	0.91	0.95
rs12984621	41732727	− 0.15	0.68	0.82	− 0.14	0.41	0.60	− 0.23	0.25	0.44
rs4802112	41734490	0.89	0.02	0.11	0.04	0.84	0.92	0.34	0.09	0.25
rs4803449	41734666	1.02	0.005	*0.05*	0.17	0.31	0.50	0.47	0.01	0.10
rs76249126	41737410	− 1.22	0.06	0.22	− 0.28	0.36	0.56	− 0.71	0.04	0.18
rs75955910	41737414	− 0.65	0.32	0.50	− 0.52	0.08	0.24	− 0.54	0.11	0.26
rs7246896	41738212	0.11	0.86	0.92	0.22	0.42	0.60	0.18	0.57	0.74
rs77287588	41739574	− 0.48	0.29	0.49	− 0.22	0.29	0.49	− 0.27	0.27	0.46
rs4802114	41741278	0.36	0.32	0.50	0.07	0.66	0.81	0.14	0.47	0.64
rs4637024	41743454	− 0.76	0.13	0.29	0.02	0.95	0.96	− 0.36	0.17	0.34
rs3786555	41748153	0.70	0.10	0.25	− 0.23	0.24	0.44	0.13	0.55	0.72
rs2304234	41748753	0.51	0.17	0.34	0.00	0.99	0.99	0.14	0.48	0.64
rs55841050	41750550	1.43	0.04	0.19	0.80	0.01	0.10	0.94	0.01	0.10
rs12983027	41753634	0.79	0.07	0.22	− 0.18	0.36	0.56	0.18	0.43	0.61
rs12978323	41756038	0.89	0.03	0.15	0.09	0.63	0.79	0.33	0.13	0.28
rs116056574	41759637	0.10	0.77	0.88	0.12	0.45	0.63	0.02	0.92	0.95
rs35546772	41764758	1.12	0.07	0.22	0.05	0.86	0.92	0.43	0.18	0.34

SNP data was only available for a subset of subjects. SNPs were modeled as ordinal variables (0 = major allele, 1 = heterozygote, and 2 = minor allele), and models were adjusted for child’s sex, admixture, and gestational age. Beta values are showing the percent change in methylation per one unit increase in SNP. Tagging SNPs were defined with a pair tag *r* ^2^ > 0.8 in Haploview with all CHS samples (*N* = 3845). FDR was used to adjust for all tests performed at the 3 methylation averages (28 × 3 tests). Significant FDR-adjusted *p* values (< 0.05) are marked in italics

*TSS* transcription start site

^a^Average of cg10564498, cg03247049, cg12722469, cg02372201, cg19848291, and cg14892768

^b^Average of cg27579501, cg00360107, cg19270050, cg24901063, and cg26521562

^c^Average of all 12 CpG sites

**Table 5:** Association between DNA methylation levels at selected CpG sites and gene polymorphisms in *AXL* in the replication population (*N* = 728)

		cg10564498 (location: 41724653)			cg12722469 (location: 41725053)			cg00360107 (location: 41731934)		
RS Number	Location	*β*	*p*	Adjusted *p*	*β*	*p*	Adjusted *p*	*β*	*p*	Adjusted *p*
rs2301235	41724671	0.94	0.002	*0.004*	0.39	0.11	0.15	− 0.07	0.57	0.63
rs2569692	41724687	1.75	1.2E*−*07	*7.7E−07*	1.83	8.8E*−*12	*3.7E−10*	0.37	0.004	*0.007*
rs28364580	41724885	1.57	5.6E*−*07	*2.9E−06*	1.66	8.2E*−*11	*2.3E−09*	0.31	0.01	*0.02*
rs1709122	41725754	1.80	4.2E*−*12	*3.5E−10*	1.31	7.9E*−*10	*1.7E−08*	0.19	0.06	0.08
rs4803446	41726167	1.43	2.0E*−*07	*1.2E−06*	1.02	6.4E*−*06	*2.5E−05*	0.21	0.05	0.07
rs2271546	41727197	2.21	2.8E*−*08	*2.3E−07*	1.93	2.5E*−*09	*4.1E−08*	0.33	0.03	*0.05*
rs186235601	41728703	1.91	5.0E*−*04	*0.001*	2.13	1.9E*−*06	*8.5E−06*	0.57	0.007	*0.01*
rs10409940	41728765	1.46	2.1E*−*08	*2.0E−07*	1.18	3.0E*−*08	*2.3E−07*	0.18	0.07	0.10
rs11083613	41729505	0.10	0.74	0.77	0.06	0.79	0.79	0.08	0.46	0.53
rs4803447	41731175	0.57	0.22	0.27	1.04	0.006	*0.01*	0.38	0.03	*0.05*
rs59423102	41731749	0.57	0.16	0.20	0.58	0.08	0.10	0.06	0.70	0.75
rs12462203	41732423	0.77	0.002	*0.004*	0.81	5.1E*−*05	*1.7E−04*	0.47	5.1E*−*07	*2.8E−06*
rs12984621	41732727	0.55	0.03	*0.05*	0.58	0.006	*0.01*	0.36	1.9E*−*04	*5.6E−04*
rs4802112	41734490	0.87	9.8E*−*04	*0.002*	0.93	1.5E*−*05	*5.6E−05*	0.56	2.0E*−*08	*2.0E−07*
rs4803449	41734666	1.08	2.4E*−*05	*8.4E−05*	1.11	9.3E*−*08	*6.5E−07*	0.54	1.7E*−*08	*2.0E−07*
rs76249126	41737410	− 0.93	0.03	*0.05*	− 0.49	0.17	0.21	− 0.08	0.62	0.67
rs75955910	41737414	− 1.42	0.003	*0.005*	− 1.34	5.7E*−*04	*0.001*	− 0.07	0.72	0.76
rs7246896	41738212	0.79	0.10	0.13	0.22	0.57	0.63	− 0.05	0.78	0.79
rs77287588	41739574	− 0.46	0.17	0.21	− 0.84	0.002	*0.005*	0.04	0.73	0.77
rs4802114	41741278	0.47	0.08	0.10	0.68	0.001	*0.003*	0.33	8.9E*−*04	*0.002*
rs4637024	41743454	− 0.32	0.39	0.45	− 0.04	0.90	0.90	− 0.09	0.54	0.61
rs3786555	41748153	0.85	0.003	*0.005*	0.79	6.9E*−*04	*0.002*	0.50	3.2E*−*06	*1.4E−05*
rs2304234	41748753	0.37	0.13	0.16	0.66	9.6E*−*04	*0.002*	0.46	7.2E*−*07	*3.6E−06*
rs55841050	41750550	0.58	0.23	0.28	1.08	0.007	*0.01*	0.57	0.002	*0.005*
rs12983027	41753634	1.03	0.001	*0.002*	1.12	1.3E*−*05	*5.0E−05*	0.48	5.2E*−*05	*1.7E−04*
rs12978323	41756038	0.99	2.1E*−*04	*5.8E−04*	1.06	1.2E*−*06	*5.6E−06*	0.59	6.4E*−*09	*8.9E−08*
rs116056574	41759637	0.77	0.003	*0.005*	0.79	1.3E*−*04	*3.9E−04*	0.35	3.5E*−*04	*9.2E−04*
rs35546772	41764758	1.31	0.006	*0.01*	1.45	2.1E*−*04	*5.8E−04*	0.69	1.4E*−*04	*4.2E−04*

SNP data was only available for a subset of subjects. SNPs were modeled as ordinal variables (0 = major allele, 1 = heterozygote, and 2 = minor allele) and models were adjusted for child’s sex, admixture and gestational age. Beta values are showing the percent changes in methylation at each CpG site per one unit increase in SNP. Tagging SNPs were defined with a pair tag *r* ^2^ > 0.8 in Haploview with all CHS samples (*N* = 3845). FDR was used to adjust for all tests performed at the 3 CpG sites (28 × 3 tests) in each study population. Significant FDR-adjusted *p* values (< 0.05) are marked in italics

**Fig. 2:**
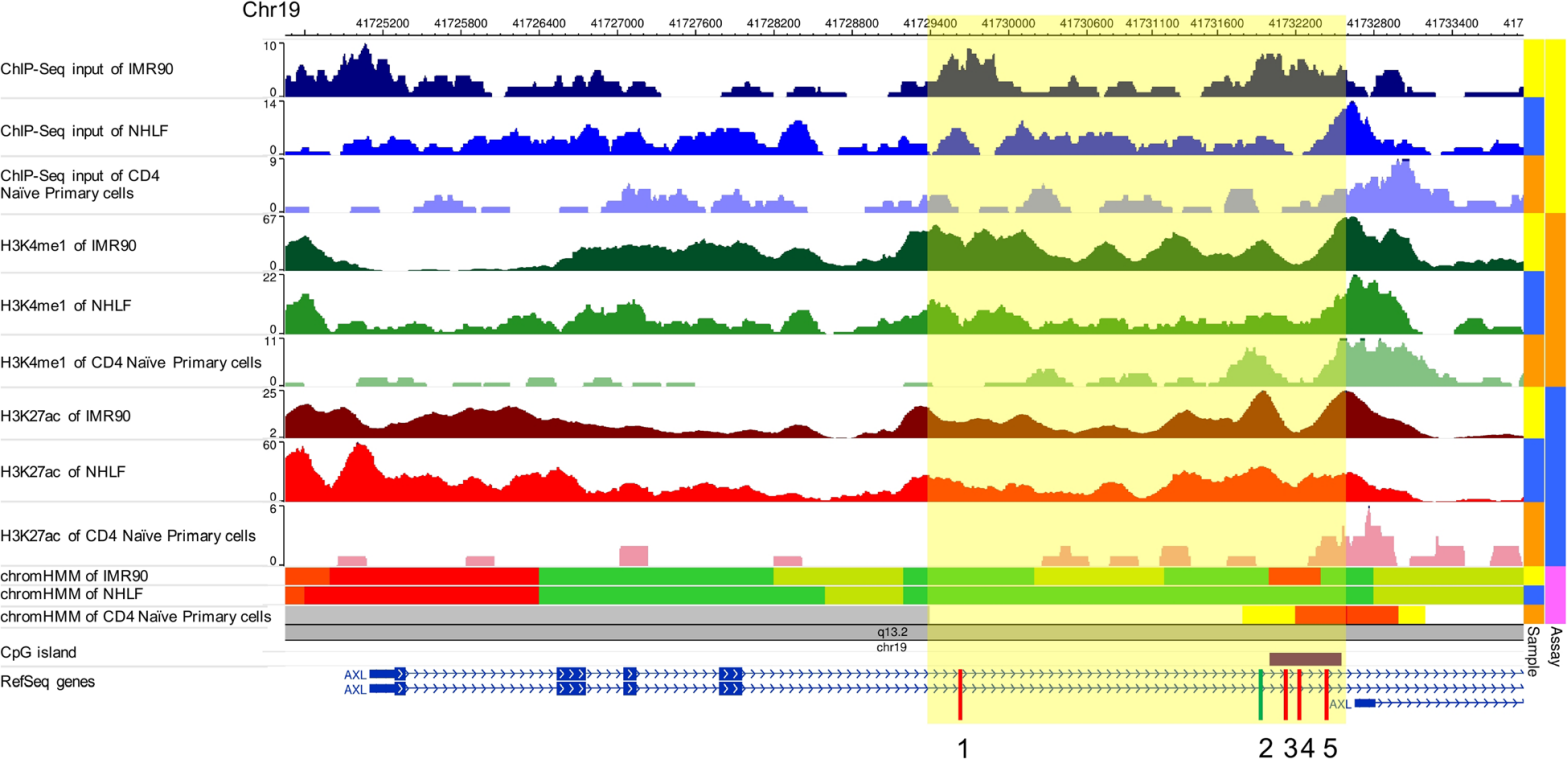
Illustration of epigenetic marks in *AXL* gene-body region (yellow box) and CpG sites (red and green bars) in multiple cell lines. This region contains a putative enhancer in IMR90 fetal lung fibroblast cells (light green in chromHMM track) and adult CD4 Naïve Primary cells (yellow in chromHMM track) and is adjacent to a putative enhancer in NHLF adult lung fibroblast cells (light green in chromHMM track). There are also transcription factor binding sites located within this region in all three cell lines from ChIP-Seq input. This region is enriched with epigenetic marks for poised enhancer (indicated by H3K4me1), active enhancer (indicated by H3K27ac), and active transcription (green in chromHMM track). CpG site 1: cg27579501; CpG site 2: cg00360107; CpG site 3: cg19270050; CpG site 4: cg24901063; CpG site 5: cg26521562. IMR90: fetal lung fibroblast cell; NHLF: normal adult lung fibroblast cell; CD4 Naïve Primary cells: obtained from adult blood

Lastly, we used the WashU EpiGenome Browser to conduct an in silico investigation of epigenetic regulatory traits in tissues and cell types relevant to our fetal programming hypothesis and potential involvement of immune cells. We contrasted histone marks, regulatory regions, and transcription factor binding sites in the IMR90 fetal lung cell line, adult lung fibroblast cells, and adult CD4 naïve primary cells (Fig. 2). The CpG loci evaluated in the *AXL* gene body region are located adjacent to enhancers in both fetal and adult lung fibroblast cells, and adult CD4 naïve primary cells (light green and yellow in chromHMM tracks). The gene-body region under investigation also contains transcription factor binding sites, indicated by peaks from ChIP-Seq input, and marks for active transcription (green in chromHMM tracks). There are more transcription factor binding sites and active histone marks associated with this region in fetal lung than in adult lung and blood, suggesting the active transcription and regulation of *AXL* in fetal lung. Additionally, the combination of H3K4me1 and H3K27ac, which marks active enhancers [59, 60], was observed in the same genomic position (CpG sites cg00360107-cg26521562) in both fetal and adult lung fibroblast cells, suggesting this region may act as an enhancer throughout life. The epigenetic marks in CD4 naïve primary cells were fewer, although the pattern of H3K4me1 in particular was generally consistent with lung cells and suggests that these epigenetic marks observed in blood may reflect patterns in fetal and adult lung.

## Discussion

Our results show that average methylation in *AXL* at birth was associated with higher risk for asthma-related phenotypes in childhood, especially wheezing. The effects of average *AXL* methylation on wheezing symptoms were magnified in girls compared to boys. One CpG locus, cg00360107, which was inversely correlated with its nearest neighbors, was associated with marginally significantly reduced wheezing risk, and the result was replicated by Pyrosequencing in a separate population of 1038 CHS subjects.

The *AXL* CpG region showing the strongest association with wheeze is located in a region of the gene body harboring histone marks for active transcription and enhancers in fetal and adult lung cells and CD4 immune cells. This region was predicted from the UCSC genome browser [61, 62] to have binding sites for the transcription factor IRF7 (interferon regulatory factor 7), which is involved in transcriptional activation of virus-inducible cellular genes, the transcriptional activator ISGF3 (interferon-stimulated gene factor 3), and AP-1 (activator protein 1) that regulates gene expression in response to a variety of pathogenic stimuli [63–65]. Alterations in CpG methylation levels in this region during fetal development may modify the transcriptional activity of *AXL* and the binding of transcription factors in response to stimuli, particularly in the lung.

*AXL* has been well characterized in the pathogenesis of numerous cancers and cardiovascular events [66–71] but is rarely addressed in asthma. Key elements in asthma pathogenesis include the accumulation of polarized CD4^+^ T helper (T_H_)2 cells and exaggeration of pro-inflammatory T_H_2 cells over the infection-fighting T_H_1 cells in the T cell repertoire, accompanied by an upregulation of the T_H_2 inflammatory cytokines [72]. In the key antigen-presenting cells including dendritic cells (DCs) and macrophages, *AXL* and other TAM proteins function to inhibit production of pro-inflammatory cytokines that are induced by Toll-like receptors (TLRs), while activating the inflammation-inhibitory genes encoding the suppressor of cytokine signaling (SOCS) 1 and 3 [29, 73]. Taken together, these concepts illuminate a carefully regulated feedback control process that switches a pro-inflammatory signaling complex to one that inhibits inflammation. Many of the genes inhibited or activated by *AXL* in this process are involved in asthma pathogenesis.

This information suggests that *AXL* signaling may be associated with the suppression of inflammatory responses and lower risk for asthma and related phenotypes. In our study, we observed average DNA methylation at *AXL* was positively associated with wheezing symptoms. The association was stronger in girls, where a 1% increase in average methylation was associated with an 88% increase in risk of wheezing symptoms while no effect was seen in boys. Previous research has reported sex-specific associations between DNA methylation and various health outcomes including autoimmune diseases [74–76], although the mechanisms behind these are unclear. The association between *AXL* methylation and higher risk for wheezing symptoms was observed as early as the first few days of life, indicating that methylation status of *AXL* may be reflecting epigenetic changes programmed in utero that make the child more susceptible to symptoms in later childhood. Higher average *AXL* methylation was also associated with higher risk for childhood bronchitic symptoms, which are suggestive of chronic symptoms that may follow an illness or acute exacerbation of asthma, or chronic inflammation in the airway. However, due to the small sample size in the primary population, we were not able to detect significant associations.

Underlying genetic variants are known to influence epigenetic variation. Therefore, we evaluated SNPs in *AXL* to understand whether genetic variation influenced DNA methylation directly, to address potential confounding of observed associations between DNA methylation and asthma and wheeze risk, and to test whether SNPs independently predicted asthma and wheeze risk. Genome-wide studies have revealed quantitative trait loci (QTLs) for DNA methylation, known as methylation QTLs (metQTLs) in multiple human tissues [77–83]. MetQTLs are usually located in intergenic or intragenic regions and affect DNA methylation levels at nearby CpG sites [84]. In one study of metQTLs in human lung, the authors identified 34,304 *cis*- and 585 *trans*-metQTLs, which were enriched in CTCF-binding sites, DNaseI hypersensitivity regions, and histone marks [81]. In this study, we found that average DNA methylation in *AXL* was highly correlated with genetic variation in nearby sites acting in *cis*.

The above evidence implies that alterations in the methylation landscape of *AXL* may be attributable partially to genetic polymorphisms in nearby regions. Most of the SNPs under investigation in this paper and the reported methylation-associated SNPs were located in gene-body intragenic regions [84], suggesting an interaction between gene-body methylation and proximal genetic variants. However, none of the SNPs under investigation were implicated in asthma and related symptoms in childhood in this study and none of the SNPs acted as confounders of the observed associated between DNA methylation and asthma-related symptoms.

One of the strengths of this study is the temporal separation of DNA methylation assessment (at birth) and respiratory health outcomes assessment (at 6-7 years of age), which enables the investigation of fetal factors associated with asthma predisposition while overcoming the concern for reverse causation. However, several limitations should also be noted. First, DNA methylation of *AXL* was measured from newborn blood which is a mixed cell population. Since *AXL* is expressed at very low levels in blood [85, 86], it may not be the ideal tissue to study *AXL* gene activity. Nonetheless, methylation levels systemically altered during fetal development ought to be reflected across multiple tissues, and therefore, evaluating methylation in newborn blood can serve as a useful biomarker of early life exposure relevant to the target tissue. Indeed, the lack of *AXL* expression in cord blood, but its presence in lung tissue, coupled with our in vitro assessment of the epigenetic landscape in CD4 naïve primary cells compared to lung cells supports this notion for *AXL*. Future evidence from human- or animal-based designs is warranted to demonstrate the consistent pattern of *AXL* methylation across somatic tissues and in which tissues the methylation correlates with expression. Second, characterization of asthma and related phenotypes was based on parent-completed questionnaires, potentially introducing recall bias or misclassification bias. Lastly, although we made every effort to control for potential confounders, we cannot exclude the possibility of residual confounding by some unknown factors associated with *AXL* DNA methylation levels and asthma-related phenotypes.

## Conclusions

In conclusion, *AXL* DNA methylation at birth, which was strongly linked to underlying genetic variation, was also associated with higher risk for asthma-related phenotypes in early childhood. The effects on wheezing were stronger in girls than in boys.

## Additional file

Additional file 1: Figure S1. The association between *AXL* mRNA and average methylation at all 12 CpG sites. **Figure S2.** The association between cg10564498 methylation and genotype at each tagging SNP in the replication population. **Table S1.** List of SNPs analyzed. **Table S2.** Spearman correlation between methylation at each *AXL* CpG site in the primary population. **Table S3.** Association between DNA methylation levels at *AXL* CpG sites and risk of asthma and related symptoms in childhood in the primary study population. **Table S4.** Sensitivity analysis for adding admixture in testing the association between *AXL* DNA methylation and risk of childhood asthma and related symptoms in the primary population. **Table S5.** Sensitivity analysis for adding the top 7 principal components (PCs) of *AXL* SNPs in testing the association between *AXL* DNA methylation and risk of childhood asthma and related symptoms in the primary population. **Table S6.** Association between gene polymorphisms in *AXL* and risk of asthma and related symptoms in childhood in all CHS samples. (DOCX 3935 kb)

## Abbreviations

CHS: Children’s Health Study
CI: Confidence interval
CpG: Cytosine-guanine dinucleotide sites
DNA: Deoxyribonucleic acid
FDR: False discovery rate
HM450: HumanMethylation450 BeadChip
LD: Linkage disequilibrium
MAF: Minor allele frequency
metQTL: Methylation quantitative trait loci
NBS: Newborn bloodspot
NEST: Newborn Epigenetic STudy
NHLF: Normal human lung fibroblasts
OR: Odds ratio
PC: Principal component
PCR: Polymerase chain reaction
SNP: Single-nucleotide polymorphism
TCGA: The Cancer Genome Atlas
T_H_1: Type 1 T helper;
T_H_2: Type 2 T helper
TSS: Transcription start site
