# Supplementary material for: Epigenetic regulation of AXL and risk of childhood asthma symptoms
Source: Clin Epigenetics. 2017 Nov 7;9:121. doi: 10.1186/s13148-017-0421-8 (PMC5688797; doi:10.1186/s13148-017-0421-8)
Supplement: Additional file 1: Figure S1. — The association between AXL mRNA and average methylation at all 12 CpG sites. Figure S2. The association between cg10564498 methylation and genotype at each tagging SNP in the replication population. Table S1. List of SNPs analyzed. Table S2. Spearman correlation between methylation at each AXL CpG site in the primary population. Table S3. Association between DNA methylation levels at AXL CpG sites and risk of asthma and related symptoms in childhood in the primary study population. Table S4. Sensitivity analysis for adding admixture in testing the association between AXL DNA methylation and risk of childhood asthma and related symptoms in the primary population. Table S5. Sensitivity analysis for adding the top 7 principal components (PCs) of AXL SNPs in testing the association between AXL DNA methylation and risk of childhood asthma and related symptoms in the primary population. Table S6. Association between gene polymorphisms in AXL and risk of asthma and related symptoms in childhood in all CHS samples. (DOCX 3935 kb) [file 13148_2017_421_MOESM1_ESM.docx]

**Epigenetic regulation of *AXL* and risk of childhood asthma symptoms**

Lu Gao, Joshua Millstein, Kimberly D. Siegmund, Louis Dubeau, Rachel Maguire, Frank D. Gilliland, Susan K. Murphy, Cathrine Hoyo, Carrie V. Breton

**Additional File 1**

**Figure S1.** The association between *AXL* mRNA and average methylation at all 12 CpG sites, using 29 TCGA histologically normal lung tissue samples with HM450 methylation and RNA sequencing data. Spearman correlation coefficients and p-values are shown.


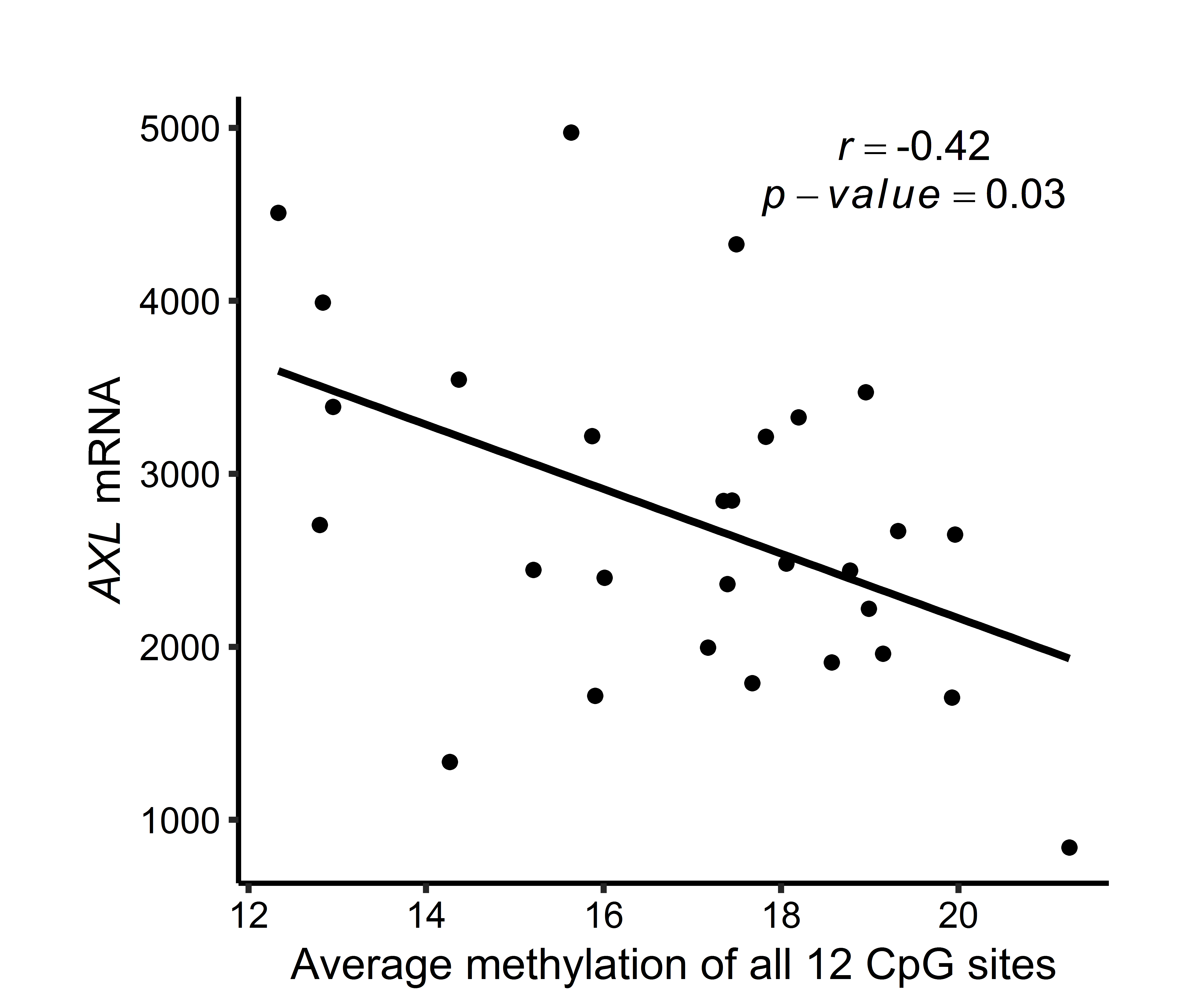


**Figure S2.** The association between cg10564498 methylation and genotype at each tagging SNP in the replication population (N=728). Unadjusted -log(p-value) are displayed, with up-triangle and down-triangle indicating positive and negative associations, respectively. Linkage disequilibrium (LD) heatmap of tagging SNPs is shown with color scheme of the r-square.


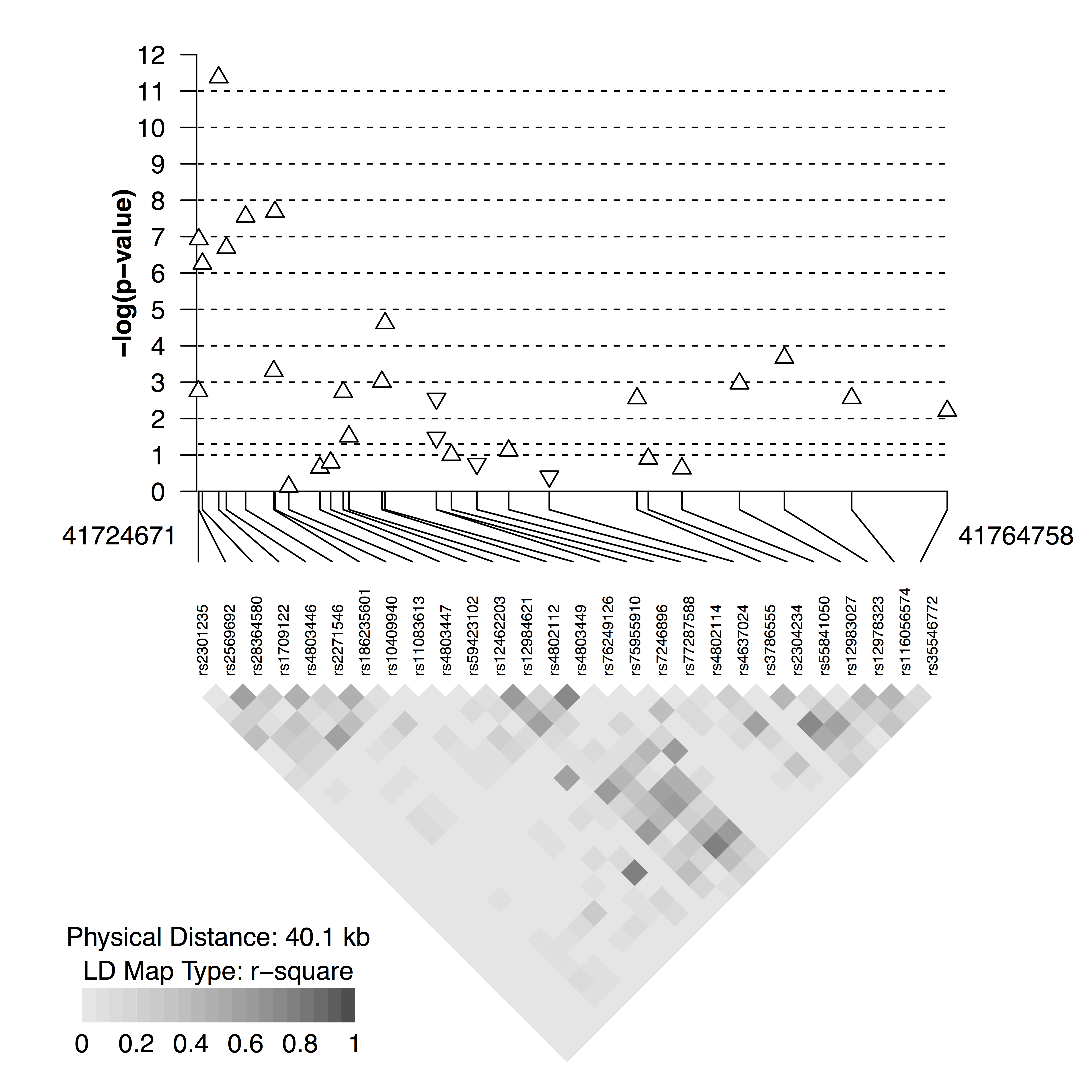


| **Table S1. List of SNPs analyzed** | | | | | |
| --- | --- | --- | --- | --- | --- |
| RS number | Location (hg19) | MAF | Tag^a^ | Tagged SNPs | eQTL tissue^b^ |
| rs2301235 | 41724671 | 0.18 | Yes | rs2301235, rs2301236 |  |
| rs2569692 | 41724687 | 0.16 | Yes | rs2569692 |  |
| rs2301236 | 41724820 | 0.18 | No |  |  |
| rs28364580 | 41724885 | 0.19 | Yes | rs28364580 |  |
| rs1654648 | 41725752 | 0.39 | No |  |  |
| rs1709122 | 41725754 | 0.38 | Yes | rs1709122, rs1709121, rs1654648 |  |
| rs1709121 | 41725790 | 0.41 | No |  |  |
| rs4803446 | 41726167 | 0.27 | Yes | rs4803446 |  |
| rs2271546 | 41727197 | 0.10 | Yes | rs2271546, rs10409443 |  |
| rs10409443 | 41728539 | 0.11 | No |  |  |
| rs186235601 | 41728703 | 0.06 | Yes | rs186235601 |  |
| rs10409940 | 41728765 | 0.39 | Yes | rs10409940 |  |
| rs11083613 | 41729505 | 0.22 | Yes | rs11083613 |  |
| rs4803447 | 41731175 | 0.09 | Yes | rs4803447, rs73043273, rs8109440, rs12459929 |  |
| rs59423102 | 41731749 | 0.10 | Yes | rs59423102 |  |
| rs12462203 | 41732423 | 0.40 | Yes | rs12462203, rs6508974, rs12978098, rs12980248, rs4802111, rs4803448, rs7246525, rs4802113, rs12979764, rs12459223, rs67033402 |  |
| rs12459929 | 41732610 | 0.09 | No |  |  |
| rs73043273 | 41732688 | 0.09 | No |  |  |
| rs12984621 | 41732727 | 0.30 | Yes | rs12984621 |  |
| rs6508974 | 41733145 | 0.39 | No |  |  |
| rs8109440 | 41733355 | 0.09 | No |  |  |
| rs4802111 | 41734059 | 0.40 | No |  |  |
| rs4802112 | 41734490 | 0.28 | Yes | rs12973055, rs4802112, rs4803451, rs4802115 | Tibial artery, subcutaneous adipose |
| rs4803448 | 41734560 | 0.41 | No |  |  |
| rs4803449 | 41734666 | 0.33 | Yes | rs4803449 | Tibial artery, subcutaneous adipose |
| rs12978098 | 41734845 | 0.41 | No |  |  |
| rs12979764 | 41734958 | 0.41 | No |  |  |
| rs12980248 | 41735115 | 0.41 | No |  |  |
| rs79855742 | 41737344 | 0.08 | No |  |  |
| rs76249126 | 41737410 | 0.08 | Yes | rs73931459, rs79855742, rs76249126, rs77033536 |  |
| rs75955910 | 41737414 | 0.07 | Yes | rs75955910 |  |
| rs12973055 | 41737851 | 0.26 | No |  |  |
| rs12973061 | 41737854 | 0.34 | No |  |  |
| rs7246525 | 41737996 | 0.41 | No |  |  |
| rs7246896 | 41738212 | 0.07 | Yes | rs7246896, rs73043294 |  |
| rs12459223 | 41738533 | 0.40 | No |  |  |
| rs73043294 | 41738716 | 0.07 | No |  |  |
| rs66841352 | 41739180 | 0.33 | No |  |  |
| rs67033402 | 41739254 | 0.41 | No |  |  |
| rs74816457 | 41739534 | 0.33 | No |  |  |
| rs77287588 | 41739574 | 0.16 | Yes | rs77287588 |  |
| rs73931459 | 41740639 | 0.08 | No |  |  |
| rs4802113 | 41740895 | 0.41 | No |  |  |
| rs4802114 | 41741278 | 0.33 | Yes | rs4802114 |  |
| rs7256873 | 41742308 | 0.17 | No |  |  |
| rs4637024 | 41743454 | 0.12 | Yes | rs4637024 |  |
| rs3786556 | 41744831 | 0.17 | No |  |  |
| rs2304235 | 41745414 | 0.17 | No |  |  |
| rs12982872 | 41746535 | 0.17 | No |  |  |
| rs3786555 | 41748153 | 0.21 | Yes | rs3786555 | Tibial artery, transformed fibroblast cells |
| rs2304234 | 41748753 | 0.37 | Yes | rs74816457, rs200156351, rs116039364, rs2304234, rs71337582, rs12973061, rs66841352, rs201282430, rs201384697, rs114806364 | Tibial artery |
| rs55841050 | 41750550 | 0.08 | Yes | rs12459996, rs55841050, rs137991784, rs73045231, rs139833223, rs17251589, rs73045226, rs12461203, rs55994820, rs73045223, rs144025261 |  |
| rs137991784 | 41751520 | 0.08 | No |  |  |
| rs12972779 | 41752127 | 0.17 | No |  |  |
| rs4803451 | 41752635 | 0.28 | No |  | Tibial artery, subcutaneous adipose, esophagus, lung |
| rs201384697 | 41752731 | 0.36 | No |  |  |
| rs200156351 | 41752732 | 0.32 | No |  |  |
| rs201282430 | 41752733 | 0.32 | No |  |  |
| rs71337582 | 41752736 | 0.32 | No |  |  |
| rs116039364 | 41752738 | 0.32 | No |  |  |
| rs114806364 | 41752739 | 0.32 | No |  |  |
| rs12983027 | 41753634 | 0.16 | Yes | rs12977563, rs35310790, rs12972779, rs3786556, rs11880729, rs11879429, rs2304232, rs11879435, rs2304235,rs4591267, rs12983027, rs1946613, rs7256873, rs12982872, rs2304231 |  |
| rs4802115 | 41753782 | 0.28 | No |  | Tibial artery, subcutaneous adipose, esophagus |
| rs11882467 | 41754217 | 0.27 | No |  | Tibial artery, subcutaneous adipose, esophagus, lung |
| rs11880729 | 41755238 | 0.14 | No |  |  |
| rs34772093 | 41755357 | 0.06 | No |  |  |
| rs139833223 | 41755390 | 0.08 | No |  |  |
| rs12978323 | 41756038 | 0.26 | Yes | rs1946612, rs12980267, rs7250883, rs11882467, rs12978323 | Tibial artery, lung, skeletal muscle, esophagus, subcutaneous adipose |
| rs17251589 | 41756085 | 0.08 | No |  |  |
| rs12977563 | 41756503 | 0.14 | No |  |  |
| rs144025261 | 41756563 | 0.08 | No |  |  |
| rs12459996 | 41756906 | 0.08 | No |  |  |
| rs11879429 | 41757444 | 0.14 | No |  |  |
| rs11879435 | 41757603 | 0.14 | No |  |  |
| rs55994820 | 41757707 | 0.08 | No |  |  |
| rs35310790 | 41758047 | 0.14 | No |  |  |
| rs73045223 | 41759474 | 0.08 | No |  |  |
| rs116056574 | 41759637 | 0.36 | Yes | rs116056574 |  |
| rs7250883 | 41760033 | 0.22 | No |  | Tibial artery |
| rs73045226 | 41760913 | 0.08 | No |  |  |
| rs77033536 | 41762000 | 0.07 | No |  |  |
| rs2304232 | 41762525 | 0.14 | No |  |  |
| rs2304231 | 41762670 | 0.14 | No |  |  |
| rs12461203 | 41764568 | 0.08 | No |  |  |
| rs35546772 | 41764758 | 0.06 | Yes | rs35546772, rs34772093 |  |
| rs12980267 | 41765229 | 0.22 | No |  | Tibial artery |
| rs4591267 | 41765407 | 0.14 | No |  |  |
| rs73045231 | 41766978 | 0.08 | No |  |  |
| rs1946613 | 41767785 | 0.14 | No |  |  |
| rs1946612 | 41767987 | 0.22 | No |  | Tibial artery |
| ^a^ Defined with a pair tag r^2^>0.8 in Haploview with all CHS samples (N=3845). | | | | | |
| ^b^ Data Source: GTEx Analysis Release V6p (dbGaP Accession phs000424.v6.p1) | | | | | |

| **Table S2. Spearman correlation between methylation at each *AXL* CpG site in the primary population (N=246)** | | | | | | | | | | | | | |
| --- | --- | --- | --- | --- | --- | --- | --- | --- | --- | --- | --- | --- | --- |
| Region | CpG | cg10564498 | cg03247049 | cg12722469 | cg02372201 | cg19848291 | cg14892768 | cg27579501 | cg00360107 | cg19270050 | cg24901063 | cg26521562 | cg20964856 |
| Promoter | cg10564498 | 1.00 | 0.57* | 0.55* | 0.30* | 0.15* | 0.12 | -0.01 | -0.14* | -0.01 | 0.01 | -0.08 | -0.17* |
|  | cg03247049 |  | 1.00 | 0.51* | 0.30* | 0.26* | 0.11 | -0.01 | -0.11 | -0.02 | -0.01 | -0.10 | -0.16* |
|  | cg12722469 |  |  | 1.00 | 0.43* | 0.24* | 0.32* | 0.08 | 0.01 | -0.08 | 0.05 | 0.09 | -0.07 |
|  | cg02372201 |  |  |  | 1.00 | 0.15* | 0.37* | 0.17* | 0.02 | -0.18* | -0.01 | 0.01 | 0.04 |
|  | cg19848291 |  |  |  |  | 1.00 | 0.31* | 0.16* | -0.13* | 0.27* | 0.11 | -0.35* | -0.35* |
|  | cg14892768 |  |  |  |  |  | 1.00 | 0.11 | 0.03 | -0.15* | -0.06 | 0.04 | 0.04 |
| Gene-body | cg27579501 |  |  |  |  |  |  | 1.00 | -0.14* | 0.01 | 0.09 | -0.15* | -0.18* |
|  | cg00360107 |  |  |  |  |  |  |  | 1.00 | -0.14* | -0.14* | 0.13* | 0.16* |
|  | cg19270050 |  |  |  |  |  |  |  |  | 1.00 | 0.10 | -0.30* | -0.26* |
|  | cg24901063 |  |  |  |  |  |  |  |  |  | 1.00 | -0.19* | -0.15* |
|  | cg26521562 |  |  |  |  |  |  |  |  |  |  | 1.00 | 0.37* |
| 3' UTR | cg20964856 |  |  |  |  |  |  |  |  |  |  |  | 1.00 |
| * p-value < 0.05 | | | | | | | | | | | | | |
| *Definition of abbreviations:* UTR=untranslated region | | | | | | | | | | | | | |

| **Table S3. Association between DNA methylation levels at *AXL* CpG sites and risk of asthma and related symptoms in childhood in the primary study population (N=246)^a^** | | | | | | | | | | | | | | | | | | | | | | | | |
| --- | --- | --- | --- | --- | --- | --- | --- | --- | --- | --- | --- | --- | --- | --- | --- | --- | --- | --- | --- | --- | --- | --- | --- | --- |
|  | cg10564498 | | cg03247049 | | cg12722469 | | cg02372201 | | cg19848291 | | cg14892768 | | cg27579501 | | cg00360107 | | cg19270050 | | cg24901063 | | cg26521562 | | cg20964856 | |
| **Distance to TSS (bp)** | -455 | | -210 | | -55 | | 44 | | 94 | | 224 | | 4549 | | 6826 | | 7015 | | 7113 | | 7360 | | 42561 | |
| **Mean methylation (%)** | 26.71 | | 21.44 | | 17.05 | | 10.13 | | 13.55 | | 45.11 | | 89.36 | | 6.68 | | 11.96 | | 9.61 | | 13.16 | | 65.00 | |
|  | OR | P | OR | P | OR | P | OR | P | OR | P | OR | P | OR | P | OR | P | OR | P | OR | P | OR | P | OR | P |
| **Ever MD-diagnosed asthma** |  |  |  |  |  |  |  |  |  |  |  |  |  |  |  |  |  |  |  |  |  |  |  |  |
| Overall | 1.02 | 0.28 | 1.10 | 0.06 | 1.05 | 0.24 | 0.92 | 0.65 | 1.24 | **0.04** | 0.99 | 0.81 | 0.88 | 0.21 | 0.73 | **0.04** | 1.05 | 0.18 | 0.92 | 0.50 | 1.03 | 0.59 | 0.89 | 0.19 |
| By sex |  |  |  |  |  |  |  |  |  |  |  |  |  |  |  |  |  |  |  |  |  |  |  |  |
| Boys | 1.04 | 0.11 | 1.10 | 0.20 | 1.08 | 0.24 | 1.16 | 0.55 | 1.35 | **0.05** | 0.99 | 0.91 | 0.95 | 0.73 | 0.72 | 0.16 | 1.04 | 0.44 | 0.81 | 0.28 | 1.05 | 0.38 | 0.91 | 0.39 |
| Girls | 0.99 | 0.80 | 1.11 | 0.16 | 1.03 | 0.59 | 0.75 | 0.26 | 1.13 | 0.38 | 0.99 | 0.83 | 0.83 | 0.14 | 0.73 | 0.13 | 1.05 | 0.24 | 1.00 | 0.99 | 0.99 | 0.92 | 0.88 | 0.25 |
| Interaction p-value | 0.24 | | 0.94 | | 0.59 | | 0.22 | | 0.37 | | 0.95 | | 0.48 | | 0.94 | | 0.84 | | 0.38 | | 0.46 | | 0.84 | |
| **Ever wheezing** |  |  |  |  |  |  |  |  |  |  |  |  |  |  |  |  |  |  |  |  |  |  |  |  |
| Overall | 1.03 | 0.21 | 1.04 | 0.44 | 1.07 | 0.09 | 0.98 | 0.88 | 1.16 | 0.15 | 0.98 | 0.58 | 1.11 | 0.27 | 0.78 | 0.07 | 1.10 | **0.005*** | 1.13 | 0.18 | 1.13 | **0.007*** | 1.00 | 1.00 |
| By sex |  |  |  |  |  |  |  |  |  |  |  |  |  |  |  |  |  |  |  |  |  |  |  |  |
| Boys | 0.98 | 0.59 | 1.10 | 0.28 | 1.07 | 0.38 | 1.33 | 0.22 | 1.30 | 0.15 | 1.02 | 0.83 | 1.38 | 0.07 | 1.05 | 0.87 | 1.07 | 0.26 | 1.20 | 0.38 | 1.19 | **0.008** | 0.88 | 0.32 |
| Girls | 1.07 | **0.04** | 1.01 | 0.87 | 1.08 | 0.14 | 0.78 | 0.25 | 1.10 | 0.42 | 0.96 | 0.44 | 1.03 | 0.77 | 0.69 | **0.04** | 1.11 | **0.007** | 1.11 | 0.27 | 1.09 | 0.10 | 1.06 | 0.54 |
| Interaction p-value | 0.09 | | 0.43 | | 0.94 | | 0.08 | | 0.43 | | 0.56 | | 0.15 | | 0.20 | | 0.63 | | 0.75 | | 0.23 | | 0.20 | |
| **Wheezing in the previous 12 months** |  |  |  |  |  |  |  |  |  |  |  |  |  |  |  |  |  |  |  |  |  |  |  |  |
| Overall | 1.02 | 0.60 | 0.96 | 0.61 | 0.99 | 0.93 | 0.48 | **0.04** | 0.98 | 0.89 | 0.93 | 0.31 | 1.02 | 0.87 | 0.55 | **0.04** | 1.18 | **0.003*** | 1.20 | 0.14 | 1.13 | 0.11 | 1.14 | 0.32 |
| By sex |  |  |  |  |  |  |  |  |  |  |  |  |  |  |  |  |  |  |  |  |  |  |  |  |
| Boys | 0.92 | 0.13 | 1.03 | 0.84 | 0.82 | 0.10 | 0.26 | **0.04** | 1.01 | 0.98 | 0.80 | 0.10 | 0.98 | 0.92 | 0.74 | 0.37 | 1.35 | **0.002*** | 0.95 | 0.87 | 0.98 | 0.81 | 1.04 | 0.83 |
| Girls | 1.15 | **0.02** | 0.93 | 0.43 | 1.11 | 0.22 | 0.57 | 0.15 | 0.95 | 0.83 | 0.99 | 0.88 | 1.05 | 0.78 | 0.40 | **0.03** | 1.11 | 0.10 | 1.27 | 0.09 | 1.25 | **0.009** | 1.23 | 0.22 |
| Interaction p-value | **0.009** | | 0.54 | | 0.04 | | 0.23 | | 0.86 | | 0.18 | | 0.79 | | 0.23 | | 0.08 | | 0.38 | | **0.04** | | 0.46 | |
| **Bronchitic symptoms in the previous 12 months** |  |  |  |  |  |  |  |  |  |  |  |  |  |  |  |  |  |  |  |  |  |  |  |  |
| Overall | 0.99 | 0.66 | 0.98 | 0.60 | 1.06 | 0.19 | 0.80 | 0.22 | 1.13 | 0.25 | 0.97 | 0.47 | 0.97 | 0.77 | 0.75 | 0.07 | 1.10 | **0.007** | 1.06 | 0.54 | 0.98 | 0.72 | 0.92 | 0.32 |
| By sex |  |  |  |  |  |  |  |  |  |  |  |  |  |  |  |  |  |  |  |  |  |  |  |  |
| Boys | 0.96 | 0.24 | 0.99 | 0.94 | 1.02 | 0.74 | 0.72 | 0.27 | 1.24 | 0.14 | 0.94 | 0.34 | 0.96 | 0.80 | 0.80 | 0.31 | 1.16 | **0.02** | 1.06 | 0.73 | 0.92 | 0.23 | 0.91 | 0.40 |
| Girls | 1.03 | 0.46 | 0.96 | 0.52 | 1.09 | 0.14 | 0.84 | 0.46 | 1.03 | 0.85 | 0.99 | 0.92 | 0.98 | 0.86 | 0.70 | 0.12 | 1.07 | 0.12 | 1.06 | 0.61 | 1.04 | 0.47 | 0.92 | 0.49 |
| Interaction p-value | 0.18 | | 0.70 | | 0.47 | | 0.67 | | 0.31 | | 0.53 | | 0.93 | | 0.67 | | 0.30 | | 0.99 | | 0.13 | | 0.92 | |
| *Definition of abbreviations:* TSS = transcription start site | | | | | | | | | | | | | | | | | | | | | | | | |
| Odds ratios are presented for an increase in 1% of DNA methylation level at birth. For all comparisons the reference group is children not having the corresponding outcome. Significant raw p-values (<0.05) are marked in bold. FDR was used to adjust for all tests performed at 12 CpG sites for each outcome. * indicates significant p-values (<0.05) after FDR adjustment. | | | | | | | | | | | | | | | | | | | | | | | | |
| ^a^ Adjusted for child's age, sex, ethnicity, methylation plate and city of residence at study recruitment; additionally adjusted for ever had MD-diagnosed asthma for wheezing and bronchitic outcomes | | | | | | | | | | | | | | | | | | | | | | | | |
| ^b^ Average of cg10564498, cg03247049, cg12722469, cg02372201, cg19848291 and cg14892768 | | | | | | | | | | | | | | | | | | | | | | | | |
| ^c^ Average of cg27579501, cg00360107, cg19270050, cg24901063 and cg26521562 | | | | | | | | | | | | | | | | | | | | | | | | |
| ^d^ Average of all 12 CpG sites | | | | | | | | | | | | | | | | | | | | | | | | |

| **Table S4. Sensitivity analysis for adding admixture in testing the association between *AXL* DNA methylation and risk of childhood asthma and related symptoms in the primary population (N=231)** | | | | | | | | | | | | | | | | | | | | | | | | | | | | | | |
| --- | --- | --- | --- | --- | --- | --- | --- | --- | --- | --- | --- | --- | --- | --- | --- | --- | --- | --- | --- | --- | --- | --- | --- | --- | --- | --- | --- | --- | --- | --- |
|  | cg10564498 | | cg03247049 | | cg12722469 | | cg02372201 | | cg19848291 | | cg14892768 | | cg27579501 | | cg00360107 | | cg19270050 | | cg24901063 | | cg26521562 | | cg20964856 | | Average of near-TSS CpG sites^c^ | | Average of gene body CpG sites^d^ | | Average of all 12 CpG sites^e^ | |
| **Distance to TSS (bp)** | -455 | | -210 | | -55 | | 44 | | 94 | | 224 | | 4549 | | 6826 | | 7015 | | 7113 | | 7360 | | 42561 | |  | |  | |  | |
|  | OR | P | OR | P | OR | P | OR | P | OR | P | OR | P | OR | P | OR | P | OR | P | OR | P | OR | P | OR | P | OR | P | OR | P | OR | P |
| **Ever MD-diagnosed asthma** |  |  |  |  |  |  |  |  |  |  |  |  |  |  |  |  |  |  |  |  |  |  |  |  |  |  |  |  |  |  |
| Model 1^a^ | 1.02 | 0.37 | 1.07 | 0.18 | 1.05 | 0.33 | 0.92 | 0.67 | 1.24 | **0.05** | 1.01 | 0.83 | 0.84 | 0.12 | 0.76 | 0.09 | 1.04 | 0.28 | 0.98 | 0.88 | 1.04 | 0.49 | 0.88 | 0.16 | 1.09 | 0.22 | 1.06 | 0.68 | 1.16 | 0.25 |
| Model 2^b^ | 1.02 | 0.32 | 1.07 | 0.19 | 1.05 | 0.30 | 0.92 | 0.68 | 1.24 | **0.05** | 1.01 | 0.80 | 0.85 | 0.13 | 0.76 | 0.09 | 1.04 | 0.28 | 0.97 | 0.84 | 1.04 | 0.46 | 0.88 | 0.17 | 1.09 | 0.20 | 1.06 | 0.67 | 1.17 | 0.22 |
| **Ever wheezing** |  |  |  |  |  |  |  |  |  |  |  |  |  |  |  |  |  |  |  |  |  |  |  |  |  |  |  |  |  |  |
| Model 1^a^ | 1.03 | 0.17 | 1.02 | 0.74 | 1.06 | 0.22 | 0.93 | 0.70 | 1.12 | 0.28 | 0.97 | 0.45 | 1.10 | 0.32 | 0.78 | 0.08 | 1.11 | **0.003** | 1.14 | 0.16 | 1.11 | **0.02** | 0.97 | 0.69 | 1.08 | 0.29 | 1.75 | **0.0002** | 1.42 | **0.01** |
| Model 2^b^ | 1.02 | 0.32 | 1.01 | 0.85 | 1.07 | 0.13 | 0.99 | 0.96 | 1.12 | 0.30 | 0.97 | 0.53 | 1.11 | 0.30 | 0.83 | 0.23 | 1.13 | **0.002** | 1.10 | 0.32 | 1.12 | **0.02** | 0.97 | 0.70 | 1.07 | 0.35 | 1.83 | **0.0002** | 1.41 | **0.02** |
| **Wheezing in the previous 12 months** |  |  |  |  |  |  |  |  |  |  |  |  |  |  |  |  |  |  |  |  |  |  |  |  |  |  |  |  |  |  |
| Model 1^a^ | 1.02 | 0.61 | 0.96 | 0.52 | 0.99 | 0.86 | 0.47 | **0.04** | 0.98 | 0.90 | 0.92 | 0.31 | 1.01 | 0.94 | 0.56 | **0.04** | 1.17 | **0.005** | 1.20 | 0.15 | 1.12 | 0.11 | 1.13 | 0.38 | 0.97 | 0.75 | 1.99 | **0.003** | 1.23 | 0.30 |
| Model 2^b^ | 1.03 | 0.37 | 0.98 | 0.75 | 1.01 | 0.90 | 0.48 | **0.05** | 0.98 | 0.93 | 0.91 | 0.23 | 1.01 | 0.92 | 0.58 | 0.07 | 1.17 | **0.005** | 1.19 | 0.20 | 1.12 | 0.12 | 1.13 | 0.39 | 1.00 | 0.99 | 2.01 | **0.003** | 1.34 | 0.16 |
| **Bronchitic symptoms in the previous 12 months** |  |  |  |  |  |  |  |  |  |  |  |  |  |  |  |  |  |  |  |  |  |  |  |  |  |  |  |  |  |  |
| Model 1^a^ | 0.99 | 0.60 | 0.97 | 0.55 | 1.06 | 0.15 | 0.78 | 0.18 | 1.15 | 0.19 | 0.98 | 0.73 | 0.98 | 0.83 | 0.77 | 0.10 | 1.10 | **0.008** | 1.06 | 0.55 | 0.98 | 0.62 | 0.94 | 0.46 | 1.00 | 0.96 | 1.22 | 0.15 | 1.06 | 0.67 |
| Model 2^b^ | 0.99 | 0.58 | 0.96 | 0.45 | 1.06 | 0.16 | 0.76 | 0.14 | 1.13 | 0.27 | 0.98 | 0.62 | 0.98 | 0.82 | 0.74 | 0.08 | 1.10 | **0.008** | 1.04 | 0.67 | 0.98 | 0.68 | 0.92 | 0.35 | 0.99 | 0.86 | 1.22 | 0.15 | 1.04 | 0.78 |
| *Definition of abbreviations*: TSS = transcription start site | | | | | | | | | | | | | | | | | | | | | | | | | | | | | | |
| Ancestry data was not available for all subjects. Odds ratios are presented for an increase in 1% of DNA methylation level at birth. For all comparisons the reference group is children not having the corresponding outcome. Significant raw p-values (<0.05) are marked in bold. | | | | | | | | | | | | | | | | | | | | | | | | | | | | | | |
| ^a^ Adjusted for child's age, sex, ethnicity, methylation plate and city of residence at study recruitment; additionally adjusted for ever had MD-diagnosed asthma for wheezing and BCP outcomes | | | | | | | | | | | | | | | | | | | | | | | | | | | | | | |
| ^b^ Adjusted for child's age, sex, ethnicity, methylation plate, city of residence at study recruitment and admixture; additionally adjusted for ever had MD-diagnosed asthma for wheezing and BCP outcomes | | | | | | | | | | | | | | | | | | | | | | | | | | | | | | |
| ^c^ Average of cg10564498, cg03247049, cg12722469, cg02372201, cg19848291 and cg14892768 | | | | | | | | | | | | | | | | | | | | | | | | | | | | | | |
| ^d^ Average of cg27579501, cg00360107, cg19270050, cg24901063 and cg26521562 | | | | | | | | | | | | | | | | | | | | | | | | | | | | | | |
| ^e^ Average of all 12 CpG sites | | | | | | | | | | | | | | | | | | | | | | | | | | | | | | |

| **Table S5. Sensitivity analysis for adding the top 7 principal components (PCs) of *AXL* SNPs in testing the association between *AXL* DNA methylation and risk of childhood asthma and related symptoms in the primary population (N=165)** | | | | | | | | | | | | | | | | | | | | | | | | | | | | | | |
| --- | --- | --- | --- | --- | --- | --- | --- | --- | --- | --- | --- | --- | --- | --- | --- | --- | --- | --- | --- | --- | --- | --- | --- | --- | --- | --- | --- | --- | --- | --- |
|  | cg10564498 | | cg03247049 | | cg12722469 | | cg02372201 | | cg19848291 | | cg14892768 | | cg27579501 | | cg00360107 | | cg19270050 | | cg24901063 | | cg26521562 | | cg20964856 | | Average of near-TSS CpG sites^c^ | | Average of gene body CpG sites^d^ | | Average of all 12 CpG sites^e^ | |
| **Distance to TSS (bp)** | -455 | | -210 | | -55 | | 44 | | 94 | | 224 | | 4549 | | 6826 | | 7015 | | 7113 | | 7360 | | 42561 | |  | |  | |  | |
|  | OR | P | OR | P | OR | P | OR | P | OR | P | OR | P | OR | P | OR | P | OR | P | OR | P | OR | P | OR | P | OR | P | OR | P | OR | P |
| **Ever MD-diagnosed asthma** |  |  |  |  |  |  |  |  |  |  |  |  |  |  |  |  |  |  |  |  |  |  |  |  |  |  |  |  |  |  |
| Model 1^a^ | 1.01 | 0.61 | 1.09 | 0.14 | 1.02 | 0.65 | 0.85 | 0.44 | 1.22 | 0.11 | 1.02 | 0.66 | 0.83 | 0.13 | 0.75 | 0.11 | 1.04 | 0.28 | 0.98 | 0.89 | 1.01 | 0.87 | 0.83 | 0.08 | 1.07 | 0.36 | 1.03 | 0.84 | 1.11 | 0.45 |
| Model 2^b^ | 1.01 | 0.66 | 1.08 | 0.22 | 1.01 | 0.89 | 0.87 | 0.52 | 1.21 | 0.16 | 1.02 | 0.73 | 0.81 | 0.11 | 0.64 | **0.04** | 1.05 | 0.22 | 0.97 | 0.82 | 1.03 | 0.69 | 0.83 | 0.14 | 1.05 | 0.49 | 1.05 | 0.78 | 1.10 | 0.53 |
| **Ever wheezing** |  |  |  |  |  |  |  |  |  |  |  |  |  |  |  |  |  |  |  |  |  |  |  |  |  |  |  |  |  |  |
| Model 1^a^ | 0.98 | 0.63 | 1.04 | 0.62 | 1.00 | 1.00 | 0.76 | 0.25 | 1.10 | 0.47 | 0.94 | 0.28 | 1.28 | 0.08 | 0.71 | 0.11 | 1.17 | **0.001** | 0.90 | 0.49 | 1.13 | **0.05** | 0.86 | 0.16 | 0.96 | 0.68 | 2.03 | **0.0006** | 1.19 | 0.30 |
| Model 2^b^ | 0.98 | 0.54 | 1.02 | 0.82 | 0.98 | 0.78 | 0.76 | 0.27 | 1.07 | 0.61 | 0.94 | 0.27 | 1.31 | 0.08 | 0.68 | 0.09 | 1.17 | **0.002** | 0.88 | 0.40 | 1.15 | **0.05** | 0.83 | 0.15 | 0.94 | 0.52 | 2.09 | **0.0008** | 1.14 | 0.46 |
| **Wheezing in the previous 12 months** |  |  |  |  |  |  |  |  |  |  |  |  |  |  |  |  |  |  |  |  |  |  |  |  |  |  |  |  |  |  |
| Model 1^a^ | 0.94 | 0.26 | 0.89 | 0.21 | 0.95 | 0.52 | 0.36 | 0.07 | 1.09 | 0.73 | 1.01 | 0.94 | 1.23 | 0.37 | 0.48 | 0.10 | 1.56 | **0.007** | 0.70 | 0.23 | 1.12 | 0.30 | 0.88 | 0.55 | 0.86 | 0.29 | 3.20 | **0.007** | 1.01 | 0.97 |
| Model 2^b^ | 0.92 | 0.25 | 0.90 | 0.41 | 0.99 | 0.91 | 0.23 | 0.10 | 1.14 | 0.67 | 1.07 | 0.63 | 1.54 | 0.18 | 0.46 | 0.13 | 6.03 | 0.20 | 0.57 | 0.14 | 1.12 | 0.33 | 0.70 | 0.25 | 0.84 | 0.43 | 3.75 | **0.01** | 1.25 | 0.53 |
| **Bronchitic symptoms in the previous 12 months** |  |  |  |  |  |  |  |  |  |  |  |  |  |  |  |  |  |  |  |  |  |  |  |  |  |  |  |  |  |  |
| Model 1^a^ | 0.97 | 0.35 | 0.94 | 0.32 | 1.02 | 0.76 | 0.56 | 0.03 | 1.10 | 0.50 | 1.02 | 0.76 | 0.98 | 0.88 | 0.84 | 0.40 | 1.11 | **0.02** | 0.83 | 0.26 | 0.98 | 0.74 | 0.83 | 0.10 | 0.95 | 0.55 | 1.21 | 0.27 | 0.94 | 0.73 |
| Model 2^b^ | 0.95 | 0.19 | 0.88 | 0.10 | 0.99 | 0.87 | 0.49 | 0.02 | 1.08 | 0.60 | 1.01 | 0.84 | 0.98 | 0.90 | 0.76 | 0.23 | 1.11 | **0.04** | 0.78 | 0.15 | 0.98 | 0.72 | 0.83 | 0.12 | 0.90 | 0.27 | 1.16 | 0.41 | 0.84 | 0.36 |
| *Definition of abbreviations*: PC = principal component; SNP = single nucleotide polymorphism; TSS = transcription start site | | | | | | | | | | | | | | | | | | | | | | | | | | | | | |  |
| PC data was not available for all subjects. Odds ratios are presented for an increase in 1% of DNA methylation level at birth. For all comparisons the reference group is children not having the corresponding outcome. Significant raw p-values (<0.05) are marked in bold. | | | | | | | | | | | | | | | | | | | | | | | | | | | | | | |
| ^a^ Adjusted for child's age, sex, ethnicity, methylation plate and city of residence at study recruitment; additionally adjusted for ever had MD-diagnosed asthma for wheezing and BCP outcomes | | | | | | | | | | | | | | | | | | | | | | | | | | | | | | |
| ^b^ Adjusted for child's age, sex, ethnicity, methylation plate, city of residence at study recruitment and the top 7 PCs of *AXL* SNPs; additionally adjusted for ever had MD-diagnosed asthma for wheezing and BCP outcomes | | | | | | | | | | | | | | | | | | | | | | | | | | | | | | |
| ^c^ Average of cg10564498, cg03247049, cg12722469, cg02372201, cg19848291 and cg14892768 | | | | | | | | | | | | | | | | | | | | | | | | | | | | | | |
| ^d^ Average of cg27579501, cg00360107, cg19270050, cg24901063 and cg26521562 | | | | | | | | | | | | | | | | | | | | | | | | | | | | | | |
| ^e^ Average of all 12 CpG sites | | | | | | | | | | | | | | | | | | | | | | | | | | | | | | |

| **Table S6. Association between gene polymorphisms in *AXL* and risk of asthma and related symptoms in childhood in all CHS samples (N=3845)^a^** | | | | | | | | | | | | |
| --- | --- | --- | --- | --- | --- | --- | --- | --- | --- | --- | --- | --- |
|  | **Ever MD-diagnosed asthma** | | | **Ever wheezing** | | | **Wheezing in the previous 12 months** | | | **Bronchitic symptoms in the previous 12 months** | | |
| RS Number | OR | P | Adjusted P | OR | P | Adjusted P | OR | P | Adjusted P | OR | P | Adjusted P |
| rs2301235 | 1.02 | 0.85 | 0.90 | 0.98 | 0.77 | 0.86 | 0.98 | 0.79 | 0.86 | 1.01 | 0.92 | 0.98 |
| rs2569692 | 1.01 | 0.90 | 0.90 | 0.96 | 0.57 | 0.77 | 0.89 | 0.14 | 0.38 | 0.99 | 0.87 | 0.98 |
| rs28364580 | 1.03 | 0.73 | 0.90 | 0.96 | 0.47 | 0.73 | 0.91 | 0.21 | 0.45 | 1.00 | 0.98 | 0.98 |
| rs1709122 | 0.98 | 0.81 | 0.90 | 1.01 | 0.88 | 0.91 | 0.89 | 0.08 | 0.38 | 0.91 | 0.13 | 0.50 |
| rs4803446 | 0.98 | 0.78 | 0.90 | 0.97 | 0.61 | 0.77 | 0.91 | 0.16 | 0.38 | 0.95 | 0.47 | 0.72 |
| rs2271546 | 1.04 | 0.66 | 0.90 | 0.92 | 0.34 | 0.60 | 0.86 | 0.12 | 0.38 | 0.99 | 0.91 | 0.98 |
| rs186235601 | 0.97 | 0.81 | 0.90 | 0.90 | 0.33 | 0.60 | 0.88 | 0.35 | 0.52 | 0.81 | 0.12 | 0.50 |
| rs10409940 | 1.03 | 0.66 | 0.90 | 1.03 | 0.63 | 0.77 | 0.93 | 0.26 | 0.46 | 0.92 | 0.18 | 0.50 |
| rs11083613 | 1.05 | 0.53 | 0.90 | 1.08 | 0.23 | 0.60 | 1.14 | 0.08 | 0.38 | 0.99 | 0.93 | 0.98 |
| rs4803447 | 0.94 | 0.57 | 0.90 | 0.89 | 0.19 | 0.60 | 1.00 | 0.98 | 0.98 | 0.94 | 0.56 | 0.78 |
| rs59423102 | 1.03 | 0.76 | 0.90 | 1.02 | 0.77 | 0.86 | 0.93 | 0.46 | 0.61 | 0.97 | 0.78 | 0.98 |
| rs12462203 | 0.90 | 0.09 | 0.37 | 0.97 | 0.52 | 0.77 | 0.94 | 0.29 | 0.47 | 0.92 | 0.18 | 0.50 |
| rs12984621 | 0.93 | 0.24 | 0.61 | 1.01 | 0.86 | 0.91 | 0.94 | 0.38 | 0.53 | 0.96 | 0.49 | 0.72 |
| rs4802112 | 0.88 | 0.06 | 0.31 | 0.89 | 0.04 | 0.52 | 0.88 | 0.06 | 0.38 | 0.90 | 0.10 | 0.50 |
| rs4803449 | 0.88 | 0.05 | 0.31 | 0.94 | 0.24 | 0.60 | 0.91 | 0.14 | 0.38 | 0.90 | 0.12 | 0.50 |
| rs76249126 | 1.04 | 0.75 | 0.90 | 1.09 | 0.35 | 0.60 | 1.13 | 0.26 | 0.46 | 1.08 | 0.48 | 0.72 |
| rs75955910 | 0.98 | 0.85 | 0.90 | 0.91 | 0.34 | 0.60 | 0.96 | 0.75 | 0.86 | 1.21 | 0.08 | 0.50 |
| rs7246896 | 1.02 | 0.88 | 0.90 | 1.09 | 0.36 | 0.60 | 0.99 | 0.92 | 0.95 | 0.92 | 0.46 | 0.72 |
| rs77287588 | 1.06 | 0.49 | 0.90 | 0.97 | 0.63 | 0.77 | 0.98 | 0.78 | 0.86 | 1.12 | 0.16 | 0.50 |
| rs4802114 | 0.92 | 0.18 | 0.57 | 0.93 | 0.18 | 0.60 | 0.94 | 0.34 | 0.52 | 0.89 | 0.06 | 0.50 |
| rs4637024 | 1.02 | 0.85 | 0.90 | 1.10 | 0.19 | 0.60 | 1.13 | 0.16 | 0.38 | 1.03 | 0.75 | 0.98 |
| rs3786555 | 0.85 | 0.04 | 0.31 | 0.94 | 0.32 | 0.60 | 0.84 | 0.02 | 0.38 | 0.94 | 0.41 | 0.72 |
| rs2304234 | 0.90 | 0.08 | 0.37 | 0.93 | 0.18 | 0.60 | 0.93 | 0.23 | 0.45 | 0.95 | 0.37 | 0.72 |
| rs55841050 | 0.93 | 0.55 | 0.90 | 0.83 | 0.06 | 0.52 | 0.97 | 0.79 | 0.86 | 0.88 | 0.28 | 0.68 |
| rs12983027 | 0.84 | 0.04 | 0.31 | 0.93 | 0.26 | 0.60 | 0.89 | 0.15 | 0.38 | 0.93 | 0.39 | 0.72 |
| rs12978323 | 0.85 | 0.03 | 0.31 | 0.87 | 0.02 | 0.51 | 0.87 | 0.05 | 0.38 | 0.91 | 0.17 | 0.50 |
| rs116056574 | 0.90 | 0.12 | 0.42 | 0.91 | 0.10 | 0.60 | 0.87 | 0.04 | 0.38 | 0.93 | 0.29 | 0.68 |
| rs35546772 | 0.85 | 0.22 | 0.61 | 1.00 | 0.98 | 0.98 | 0.93 | 0.58 | 0.74 | 0.99 | 0.96 | 0.98 |
| ^a^ SNPs were modeled as ordinal variables (0=major allele, 1=heterozygote, and 2=minor allele) and models were adjusted for child's sex, age, ethnicity and admixture. Odds ratios are presented for one unit increase in SNP. For all comparisons the reference group is children not having the corresponding outcome. Tagging SNPs were defined with a pair tag r^2^>0.8 in Haploview with all CHS samples (N=3845). FDR was used to adjust for tests performed at the 28 taggedSNPs. Significant FDR-adjusted p-values (<0.05) are marked in bold. | | | | | | | | | | | | |
